# Variants in structural cardiac genes in patients with cancer therapy-related cardiac dysfunction after anthracycline chemotherapy: a case control study

**DOI:** 10.1186/s40959-024-00231-3

**Published:** 2024-04-30

**Authors:** Hanne M. Boen, Maaike Alaerts, Inge Goovaerts, Johan B. Saenen, Constantijn Franssen, Anne Vorlat, Tom Vermeulen, Hein Heidbuchel, Lut Van Laer, Bart Loeys, Emeline M. Van Craenenbroeck

**Affiliations:** 1 Research Group Cardiovascular Diseases, GENCOR, University of Antwerp, Antwerp, Belgium; 2 Department of Cardiology, Antwerp University Hospital, Antwerp, Belgium; 3 Centrum of Medical Genetics, GENCOR, Antwerp University Hospital and University of Antwerp, Antwerp, Belgium

**Keywords:** Cardio-oncology, Cancer therapy-related cardiac dysfunction, Anthracyclines, Cardiogenetics, Risk stratification

## Abstract

**Background:**

Variants in cardiomyopathy genes have been identified in patients with cancer therapy-related cardiac dysfunction (CTRCD), suggesting a genetic predisposition for the development of CTRCD. The diagnostic yield of genetic testing in a CTRCD population compared to a cardiomyopathy patient cohort is not yet known and information on which genes should be assessed in this population is lacking.

**Methods:**

We retrospectively included 46 cancer patients with a history of anthracycline induced CTRCD (defined as a decrease in left ventricular ejection fraction (LVEF) to < 50% and a ≥ 10% reduction from baseline by echocardiography). Genetic testing was performed for 59 established cardiomyopathy genes. Only variants of uncertain significance and (likely) pathogenic variants were included. Diagnostic yield of genetic testing was compared with a matched cohort of patients with dilated cardiomyopathy (DCM, *n* = 46) and a matched cohort of patients without cardiac disease (*n* = 111).

**Results:**

Average LVEF at time of CTRCD diagnosis was 30.1 ± 11.0%. Patients were 52.9 ± 14.6 years old at time of diagnosis and 30 (65.2%) were female. Most patients were treated for breast cancer or lymphoma, with a median doxorubicin equivalent dose of 300 mg/m^2^ [112.5-540.0]. A genetic variant, either pathogenic, likely pathogenic or of uncertain significance, was identified in 29/46 (63.0%) of patients with CTRCD, which is similar to the DCM cohort (34/46, 73.9%, *p* = 0.262), but significantly higher than in the negative control cohort (47/111, 39.6%, *p* = 0.018). Variants in *TTN* were the most prevalent in the CTRCD cohort (43% of all variants). All (likely) pathogenic variants identified in the CTRCD cohort were truncating variants in *TTN*.

There were no significant differences in severity of CTRCD and in recovery rate in variant-harbouring individuals versus non-variant harbouring individuals.

**Conclusions:**

In this case-control study, cancer patients with anthracycline-induced CTRCD have an increased burden of genetic variants in cardiomyopathy genes, similar to a DCM cohort. If validated in larger prospective studies, integration of genetic data in risk prediction models for CTRCD may guide cancer treatment. Moreover, genetic results have important clinical impact, both for the patient in the setting of precision medicine, as for the family members that will receive genetic counselling.

**Supplementary Information:**

The online version contains supplementary material available at 10.1186/s40959-024-00231-3.

## Background

Anthracyclines (AC) are one of the most commonly used chemotherapeutic agents, but they are associated with significant toxicities, with cardiotoxicity being the most concerning. Up to 57% of all patients receiving AC develop subclinical cardiotoxicity and up to 9% experience a clinically significant decrease in left ventricular ejection fraction (LVEF) during or within the first year after treatment [1]. Whilst effective cancer treatment has resulted in a growing population of cancer survivors, it underscores the imperative for timely diagnosis, treatment, and ideally, prevention of cancer therapy-related cardiac dysfunction (CTRCD) [2].

Several tools for risk stratification have been developed to assess the individual risk prior to AC treatment [3, 4]. However, the development of AC-induced CTRCD is not fully explained by treatment regimen or patient characteristics alone. This complicates accurate risk assessment and deserves finetuning [2].

Recently, the presence of genetic variants in structural cardiac genes has been described in selected patients with CTRCD [5–9], pointing towards a genetic predisposition. This was confirmed in a larger retrospective and prospective study in cancer patients. In that population, truncating variants in *TTN* (*TTNtv)* were significantly more prevalent in patients developing CTRCD (10%) compared to a cohort of self-reported healthy volunteers (0.7%) [10]. In cardiomyocytes, the giant protein titin (encoded by *TTN*) operates as a bidirectional spring, determining the sarcomeric visco-elasticity and modulating passive stiffness [11]. *TTN* is the gene with the most identified causal variants, specifically *TTNtv,* in dilated cardiomyopathy, in up to 15-25% of patients [12, 13]. Truncating variants are variants that are predicted to have a substantial effect on the structure of titin, leading to an unstable transcript in contrast to missense variants, where a substitution of an amino acid occurs, but the overall structure of the transcript is unaffected [13].

Therefore, the identification of genes that are associated with AC-induced CTRCD opened new opportunities to define patients at high-risk and to prevent adverse outcomes. However, some knowledge gaps remain, limiting implication into standard of care. The comparison of the diagnostic yield of genetic testing between CTRCD and dilated cardiomyopathy (DCM) patients remains elusive. Additionally, information on which genes should be prioritized in the CTRCD population is lacking. In the context of a second-hit hypothesis, where a genetic variant may only lead to an overt phenotype after exposure to chemotherapy, the role of variants with milder pathogenic properties serving as potential genetic modifiers remains unclear. And lastly, there are no guidelines advising retrospective genetic testing in historic patients with CTRCD, so further proof of its importance for patients and their family members is needed.

In the current paper we aimed to study the genetic burden in CTRCD patients, compared to DCM patients and a negative control cohort without cardiac disease. We also assessed the prevalence and importance of variants of uncertain significance (VUS) in this population. Lastly, we evaluated the impact of a genetic diagnosis in the index on cascade screening and family members.

## Methods

### CTRCD, DCM and healthy control cohorts

In this single centre cohort study, patients with a history of CTRCD after AC chemotherapy, presenting between 1995 and 2020, were consecutively screened in a large academic heart failure and transplantation centre. Patients were eligible if they had developed CTRCD, defined as a decrease in LVEF to < 50% and a ≥ 10% reduction from baseline by echocardiography [14]. This definition complies with the current definition of moderate (LVEF < 50%) and severe (LVEF < 40%) CTRCD [2]. Both early (onset within one year after chemotherapy) and late (> 1 year after end of chemotherapy) CTRCD were included. Exclusion criteria were presence of LVEF < 50% or known relevant ischemic or valvular heart disease prior to treatment.

A positive control cohort consisted of patients with DCM defined according to the ESC cardiomyopathy definition [15], referred for genetic testing as part of standard clinical care and who were matched for age, gender and familial history of DCM.

A negative control cohort consisted of age and gender-matched patients without cardiac disease that underwent genetic testing for other indications (respectively patients with COVID or thoracic aorta aneurysmal disease). Absence of cardiac disease was verified via medical reports and echocardiography data if these were available.

The study conforms with the principles outlined in the Declaration of Helsinki [16]. The current study was approved by the local ethics committee of the University Hospital of Antwerp. All subjects gave written informed consent to participate in the study.

### Clinical evaluation

Clinical data at baseline and follow-up were retrieved from the patient files. Oncological diagnosis, cumulative dose of anthracycline, cardiovascular risk factors, comorbidities and cardiovascular function (echocardiography or gated blood pool scan if echocardiography was not available) were collected. Follow-up data included LVEF decline, LVEF recovery, cardiac transplantation and death.

### Genetic analysis

All eligible patients were offered genetic testing. Genomic DNA was extracted from EDTA blood using standard procedures (Chemagic DNA bloodkit special, Perkin Elmer, Waltham, MA, USA). Variant detection was performed using a an in-house HaloPlex targeted gene panel for next-generation sequencing (NGS) of 59 known cardiomyopathy genes (CM59-panel) [17]. Supplementary Table 1 shows the complete list of genes and their corresponding transcript included in the CM-panel. Analysis was performed of all coding exons including intron/exon transitions to a maximum of 15 intronic nucleotides. Variants were classified as benign or likely benign (class 1 and 2), variant of uncertain significance (class 3), likely pathogenic (class 4) or pathogenic (class 5) according to ACMG guidelines [18]. Only class 3, 4 and 5 variants were considered clinically actionable. All reported variants were confirmed using Sanger sequencing if they did not comply to strictly defined next generation sequencing quality criteria [17]. All variants were reviewed by a multidisciplinary cardiogenetic team (clinical geneticist, molecular geneticist, cardiologists and genetic counsellors).

Lastly, overall variant burden for the whole CM 59 panel was calculated as the sum of all variants divided over the total allele number of all genes (Variant burden_CM59_ = [sum off all variants]/[Allele number_gene1_ + …….. + allele number_gene59_]) [19]. For genes located on the X-chromosome (*EMD*; *FHL1*; *GLA*; *LAMP2* and *TAZ*) gender was taken into account for the calculation of the allele numbers. This calculation of variant burden on an allelic level, allows us to adjust for multiple variants in one patient and to compare with databases such as GnomAD. In short, an allele is defined as one (of two) versions of a DNA sequence at a given genomic location (National Human Genome Research Institute). As humans have paired chromosomes, and thus two copies of each gene, for most genes, two alleles are present. For genes located on the X chromosome, the number of alleles depends according to sex, two alleles are present in women whereas only one in men. Next to correcting for the number of alleles *per gene*, the calculation of variant burden on an allelic level also allows us to account for multiple variants on different genes in one individual.

### Cascade testing and segregation analysis

For all three cohorts, cascade screening for the presence of a class 4 or 5 variant was performed in first degree relatives. If a VUS was identified, family members were invited for genetical counselling with clinical and/or molecular segregation analysis where appropriate.

### Statistical analysis

Continuous variables are expressed as mean ± SD or median and range in case of skewed distribution.

Frequencies are expressed as numbers and percentages. Unpaired T-test was used for comparisons of continuous variables with normal distribution. Chi-square test was used for comparisons of categorical variables between cohorts. All analyses were performed using SPSS Statistics version 28 (IBM Corporation). A *p*-value of < 0.05 was considered statistically significant.

## Results

### Characteristics of the 3 patient cohorts

From the 129 screened patients with CTRCD, 46 patients fulfilled the in- and exclusion criteria and agreed to genetic testing. Patient characteristics of the three cohorts are shown in Table 1.

**Table 1:** Overview of CTRCD, DCM and negative control cohorts

	**CTRCD *n* = 46**	**DCM *n* = 46**	**Negative control *n* = 111**	**Statistics (*p*-value)** **CTRCD vs DCM**
**Demographics**				
Age at time of cardiac Dx^a^ (y)	52.9 ± 14.6	52.5 ± 14.3		*P* = 0.897
Current age (y)^a^	60.5 ± 14.0	57.5 ± - 13.5	57.4 ± 15.4	*P* = 0.705
Female^a^	30 (65.2%)	30 (65.2%)	63 (56.8%)	*P* = 1.000
**Severity of Cardiac disease**				
LVEF at time of cardiac Dx (%)	30.1 ± 11.0	30.7 ± 10.5		*P* = 0.797
Partial Recovery	19 (41.3%)	23 (50%)		*P* = 0.353
Complete Recovery	17 (37.0%)	11 (23.9%)		
LVEF after HF therapy (%)	46.6 ± 10.0	45.8 ± 9.9		*P* = 0.681
**Risk Factors**				
Family history of DCM^a^	8 (17.4%)	8 (17.4%)		*P* = 1.000
Hypercholesterolemia	19 (41.3%)	17 (37.0%)		*P* = 0.669
Arterial hypertension	24 (52.2%)	19 (41.3%)		*P* = 0.296
Obesity	14 (30.4%)	20 (43.5%)		*P* = 0.195
Diabetes Mellitus	7 (15.2%)	5 (10.9%)		*P* = 0.563
Smoker	17 (37.0%)	20 (43.5%)		*P* = 0.524
**Heart Failure Therapy**				
Beta blockers	39 (84.8%)	40 (87.0%)		*P* = 0.563
RAAS-inhibitors	31 (67.4%)	16 (34.8%)		*P* = 0.002
Aldosterone-antagonist	16 (34.8%)	22 (47.8%)		*P* = 0.172
Sacubitril/valsartan	8 (17.4%)	26 (56.5%)		***P* < 0.001**

*DCM* dilated cardiomyopathy, *CTRCD* cancer-therapy related cardiac dysfunction, *LVEF* left ventricular ejection fraction, *Dx* diagnosis, *HF* heart Failure, *RAAS* renin-aldosterone-angiotensin system. Continuous variables with a normal distribution are displayed as mean ± S.D. Categorical variables are displayed as numbers and percentage. One-way Anova was used to compare distribution of continuous variables between groups. Chi-square test was used to compare proportions between groups. Patients were matched for the variables depicted with^a^

Patients in the CTRCD cohort were predominantly female (65.2%). LVEF at time of CTRCD diagnosis was 30.1% (± 11.0) which represented a decrease of 26.6% (± 9.9) from baseline. Patients in the DCM cohort were matched for age (53 ± 14 years), gender (65.2% female) and presence of a positive family history for DCM (8/46). Mean LVEF at time of DCM diagnosis was 30.7 ± 10.5%. Presence of cardiovascular risk factors was similar as in the CTRCD group. A smaller proportion of DCM patients showed partial (rise in LVEF > 10%, but LVEF remained < 50%) or complete (LVEF > 50%) recovery after initiation of heart failure therapy compared to CTRCD patients (41.3 and 37% respectively).

Patients in the negative control cohort were age and sex -matched to the CTRCD and DCM cohort (mean age 57 ± 15 years, 56.8% female).

As shown in Table 2, CTRCD patients had been treated for lymphoma (52.2%) and/or breast cancer (43.5%) and total anthracycline dose averaged 300 mg/m^2^ doxorubicin equivalents (range 112.5-540.0 mg/m^2^). Most patients (73.9%) presented with late (median time of 7 years between AC treatment and diagnosis) and severe CTRCD (LVEF < 40%). During follow-up, 78.3% of patients showed recovery of LVEF after initiation of heart failure treatment (partial recovery in 41.3% and complete (defined as LVEF > 50%) in 37.0%). One patient received a heart transplantation and one patient died during follow-up (non-cardiovascular mortality).

**Table 2:** Patient cohort characteristics and differences between patients with a pathogenic variant (*TTN*tv), a variant of unknown significance only and no variant

	**Overall (*n* = 46)**	***TTN*tv + (*n* = 3)**	**VUS (*n* = 26)**	**Non-variant harbouring (*n* = 17)**	**Statistics**		
					***TTNt*v vs VUS**	***TTN*tv vs non**	**VUS vs non**
**Demographics**							
Current age (y)	60.5 ± 14.0	63.3 ± 7.2	59.0 ± 14.6	62.1 ± 14.3	*P* = 0.623	*P* = 0.889	*P* = 0.499
Age at time of AC treatment (y)	45.0 ± 15.5	49.7 ± 0.6	43.8 ± 16.1	45.9 ± 16.2	*P* = 0.077	*P* = 0.701	*P* = 0.674
Age at time of CTRCD diagnosis (y)	52.9 ± 14.6	56.3 ± 8.4	50.4 ± 14.5	55.9 ± 15.5	*P* = 0.498	*P* = 0.967	*P* = 0.241
Female (n,%)	30 (65.2)	2 (66.7)	17 (65.4)	11 (64.7)	*P* = 0.965	*P* = 0.948	*P* = 0.964
Time between AC treatment and onset of CTRCD (y)	7 [0-28]	3 [1–16]	4.5 [0-28]	9 [0-25]	*P* = 0.761	*P* = 0.479	***P* = 0.049**
Early onset (< 1y)	12 (26.1)	1 (33.3)	9 (34.6)	2 (11.8)	*P* = 0.965	*P* = 0.335	*P* = 0.093
**Oncological characteristics**							
Breast cancer	17 (37.0)	2 (66.7)	9 (34.6)	6 (35.2)	*P* = 0.862	*P* = 0.927	*P* = 0.829
Leukaemia	2 (4.3)	0	1 (3.8)	1 (5.9)			
Lymphoma	22 (47.8)	1 (33.3)	14 53.8)	7 (41.2)			
Sarcoma	2(4.3)	0	1 (3.8)	1 (5.9)			
BC + leukaemia	1 (2.2)	0	0	1 (5.9)			
BC + lymphoma (n,%)	2(4.3)	0	1 (3.8)	1 (5.9)			
Left sided BC	10 (50)	1 (66.6)	4 (40)	5 (62.5)	*P* = 0.787	*P* = 0.837	*P* = 0.360
Bilateral BC (n,%)	2 (10)	0 (0)	2 (20)	0 (0)			
DOX	32 (69.6)	2 (66.6)	19 (73,1)	11 (64.7)	*P* = 0.883	*P* = 0.798	*P* = 0.282
EPI	11 (23.9)	1 (33.3)	6 (23.1)	4 (23.5)			
DAUNO	1 (2.2)	0	1 (3.8)	0 (0)			
DOX + DAUNO (n,%)	2 (4.3)	0	0	2 (11.8)			
Doxorubicin equivalent dose (mg/m^2^)	300.0 [112.5-540.0]	300.0 [180.0-400.0]	300.0 [150.0-540.0]	300.0 [112.5-400]	*P* = 0.866	*P* = 0.616	*P* = 0.408
Radiotherapy (n,%)	26 (56.5)	2 (66.6)	12 (46.2)	12 (70.6)	*P* = 0.541	*P* = 0.891	*P* = 0.147
Radiotherapy to left chest (n,%)	13 (28.9)	1 (33.3)	7 (26.0)	5 (29.4)	*P* = 0.847	*P* = 0.891	*P* = 0.921
Trastuzumab (n,%)	6 (13.0)	0	4 (15.4)	2 (11.8)	*P* = 0.464	*P* = 0.531	*P* = 0.738
Hormonal therapy (n,%)	17 (37.0)	2 (66.6)	7 (26.9)	8 (47.1)	*P* = 0.159	*P* = 0.531	*P* = 0.176
**Severity of CTRCD**							
LVEF before AC (%)	56.7 ± 7.4	61.3 ± 14.5	58.0 ± 7.4	53.9 ± 5.1	*P* = 0.503	*P* = 0.470	*P* = 0.058
LVEF after AC (%)	30.1 ± 11.0	28.3 ± 15.3	30.5 ± 10.9	29.7 ± 11.1	*P* = 0.751	*P* = 0.853	*P* = 0.809
Decrease in LVEF (%)	26.6 ± 9.9	33.0 ± 6.0	27.4 ± 8.2	24.24 ± 12.2	*P* = 0.268	*P* = 0.247	*P* = 0.312
Moderate CTRCD	11 (23.9)	1 (33.3)	7 (26.9)	3 (17.6)	*P* = 0.814	*P* = 0.531	*P* = 0.481
Severe CTRCD (n,%)	35 (76.1)	2 (66.6)	19 (73.1)	14 (82.4)			
Symptomatic heart failure at Dx (n,%)	37 (80.4)	2 (66.6)	19 (73.1)	16 (94.1)	*P* = 0.814	*P* = 0.144	*P* = 0.083
Partial recovery (n,%)	19 (41.3)	3 (100)	9 34.6)	7 (41.2)	*P* = 0.093	*P* = 0.201	*P* = 0.835
Complete recovery (n,%)	17 (37.0)	0	11 42.3)	6 (35.3)			
LVEF after HF treatment (%)	46.6 ± 10.0	44.7 ± 9.2	47.4 ± 9.2	45.7 ± 10.5	*P* = 0.657	*P* = 0.878	*P* = 0.598
**Cardiovascular risk Factors**							
Arterial hypertension (n, %)	24 (52.2)	1.5 (33.3)	14 (53.8)	9 (52.9)	*P* = 0.501	*P* = 0.531	*P* = 0.954
Hypercholesterolemia (n,%)	19 (41.3)	2 (66.6)	12 (46.2)	5 (29.4)	*P* = 0.501	*P* = 0.212	*P* = 0.272
Familial history of CVD (n,%)	8 (17.4)	1 (33.3)	4 (15.4)	3 (17.6)	*P* = 0.436	*P* = 0.531	*P* = 0.844
Obesity (n,%)	14 (30.4)	1 (33.3)	8 (30.8)	5 (29.4)	*P* = 0.928	*P* = 0.891	*P* = 0.925
Diabetes Mellitus (n,%)	7 (15.2)	0	5 (19.2)	2 (11.8)	*P* = 0.404	*P* = 0.531	*P* = 0.517
Smoker (n,%)	17 (37.0)	0	11 (42.3)	6 (35.3)	*P* = 0.153	*P* = 0.219	*P* = 0.646
**Heart Failure Therapy**							
Beta blockers (n,%)	39 (84.8)	2 (66.6)	22 (84.6)	15 (78.9)	*P* = 0.436	*P* = 0.335	*P* = 0.738
RAAS-inhibitors (n,%)	31 (67.4)	2 (66.6)	20 (76.9)	9 (52.9)	*P* = 0.694	*P* = 0.660	*P* = 0.101
Aldosterone-antagonist (n,%)	16 (34.8)	2 (66.6)	6 (23.1)	8 (47.1)	*P* = 0.110	*P* = 0.531	*P* = 0.101
Sacubitril/valsartan (n,%)	8 (17.4)	0 (0)	2 (7.7)	6 (35.3)	*P* = 0.619	*P* = 0.219	*P*** = 0.023**

Continuous variables with a normal distribution are displayed as mean ± S.D. Continuous variables without normal distribution are displayed as median and range. Total anthracycline dose was calculated as doxorubicin equivalent dose with the following conversion factors: Doxorubicin 1x; Epirubicin 0.5x; Daunorubicin 0.5x; Idarubicin 2x; Mitoxantrone 2.2x

*AC* anthracycline chemotherapy, *BC* breast cancer, *CTRCD* cancer-therapy related cardiac dysfunction, *CVD* cardiovascular disease, *DAUNO* daunorubicin, *DOX* doxorubicin, *Dx* diagnosis, *EPI* epirubicin, *HF* heart failure, *LVEF* left ventricular ejection fraction, *RAAS* renin-angiotensin-aldosterone system, *TTNtv* truncating variant in titin, *VUS* variant of unknown significance

### Diagnostic yield of genetic testing in CTRCD

**Fig. 1:**
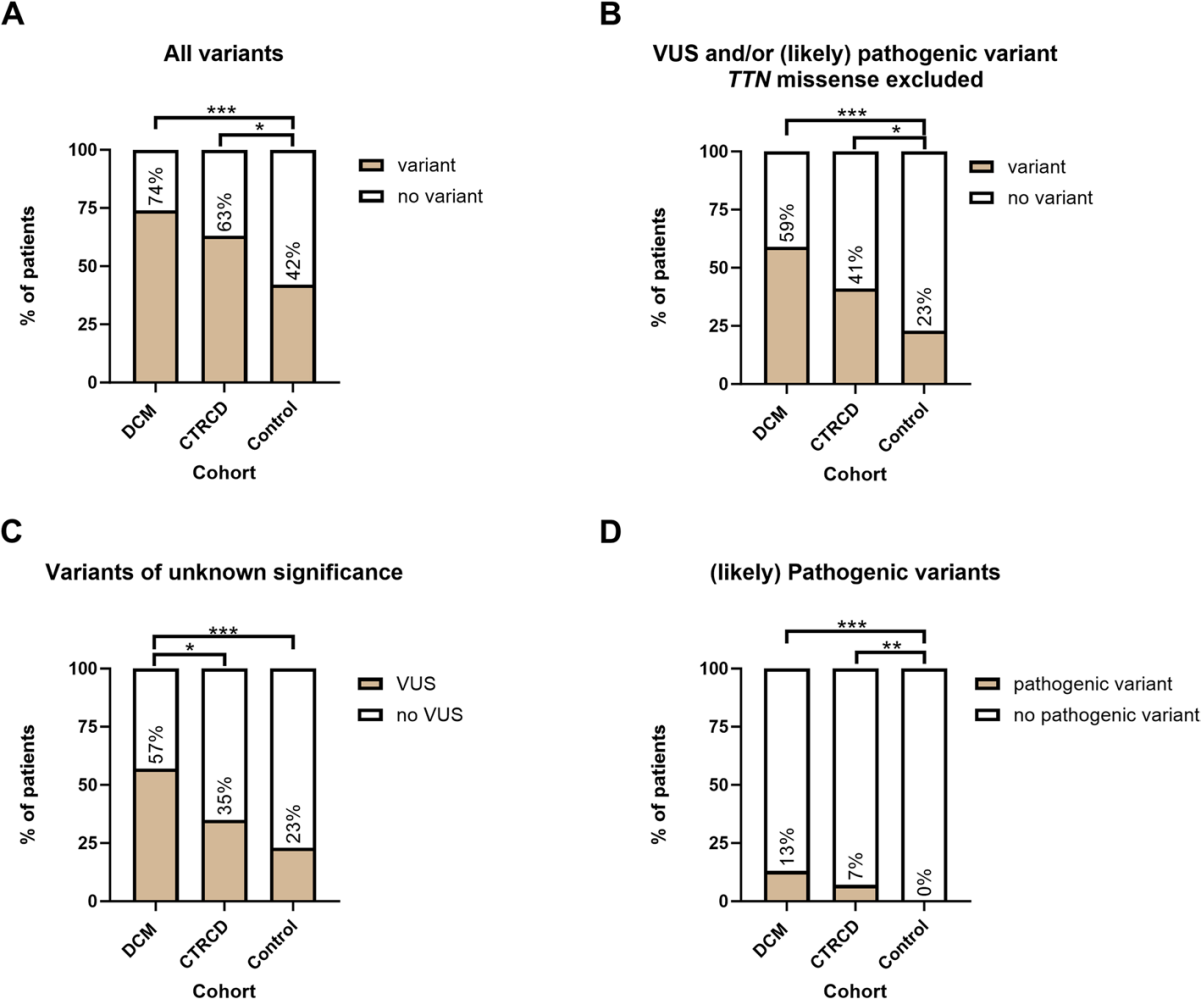
Overview of genetic yield in each cohort. Genetic yield of a 59-gene cardiomyopathy panel in a cohort of patients with DCM, CTRCD and a negative control cohort. **A** Total yield in each cohort, including *TTN* missense variants, a significant difference is present between control patients and DCM patients (*p* < 0.001) and CTRCD patients (*p* = 0.018). **B** Total yield in each cohort, without *TTN* missense variants, a significant difference is present between control patients and DCM patients (*p* < 0.001) and CTRCD patients (*p* = 0.017). **C** Prevalence of VUS in each cohort, without TTN missense variants, a significant difference is present between control patients and DCM patients (*p* < 0.001) and between DCM and CTRCD patients (*p* = 0.036), but not between CTRCD patients and controls (*p* = 0.111). **D** Prevalence of (likely) pathogenic variants in each cohort, a significant difference is present between control patients and DCM patients (*p* < 0.001) and CTRCD patients (*p* = 0.007). CTRCD: Cancer Therapy related cardiac dysfunction; DCM: dilated cardiomyopathy; VUS: variant of uncertain significance. *,**,*** respectively *p* < 0.05, 0.01,0.001

As shown in Fig. 1A, 29 patients (63%) in the CTRCD cohort carried either a VUS (*n* = 26; 56.5%) and/or a likely pathogenic variant (*n* = 3; 6.5%). The latter were all truncating variant in *TTN* (*TTN*tv).

In total, 50 variants were identified fulfilling the filtering criteria (Supplementary Table 2). Thirteen individuals harboured more than one variant (28.3%): seven patients with two variants; four patients with three variants and two patients with four variants.

When comparing variant-harbouring individuals and non-variant-harbouring individuals, no significant differences were observed in demographic, clinical and echocardiographic data. When patients were divided in severe versus moderate CTRCD (LVEF < 40% vs 40-49%) no significant differences in variant burden yield were identified. Patients with early (< 1y after treatment) onset of CTRCD (*n* = 12) more frequently harboured a variant (10; 83.3%) than patients with late onset (*n* = 19/34; 55.9%; *p* = 0.090). In one individual with early onset a likely pathogenic variant was identified (8.3%) compared to two individuals with the late onset (5.7%, *p* = 0.768).

When looking at the extremes of the spectrum, those three patients who carried a *TTNtv* did have a similar LVEF after AC (ranging from 9-45%) compared to patients without any variant (range 9–48%) and patients with a VUS only (13-48%).

Patients who carried more than one variant were not significantly worse affected than patients with only one or no variant. LVEF after AC treatment averaged 30.9 ± 10.12% in patients with more than one variant vs 29.8 ± 12.1% with only one variant and 30.6 ± 10.8% in patients without a variant (*p* = 0.408). There was no difference in recovery of LVEF after heart failure treatment either (LVEF 45.9. ± 9.9% vs 48.2 ± 10.1% vs 45.7 ± 10.5% in patients with more than one, only one or no variant respectively (*p* = 0.918)).

### Comparison with DCM cohort

In DCM, 34/46 patients carried a VUS and/or a (likely) pathogenic variant (73.9%), a burden that is similar to that of the CTRCD cohort (63% *p* = 0.262) (Fig. 1A). A (likely) pathogenic variant could be identified in 6 individuals (7 variants). A complete overview of all variants identified in the DCM cohort is provided in Supplementary Table 2.

As one patient can carry more than one variant, we next compared the variant burden on an allelic level instead of an individual level. The genetic burden in the CTRCD cohort on an allelic level was significantly lower than in the DCM cohort (0.0094 vs 0.0140; *p* = 0.023).

### Comparison with negative control cohort

In the negative control cohort, 47 patients (42.3%) carried a variant which is significantly less than the CTRCD cohort (*p* = 0.018) and DCM cohort (*p* < 0.001). No pathogenic variants were identified in the negative control cohort patients (Fig. 1D). A total of 68 variants were present in the negative control cohort, resulting in a genetic burden in the TAAD cohort on an allelic level of 0.0073 which was significantly lower than in the DCM cohort (*p* < 0.001) but not the CTRCD cohort (*p* = 0.175).

### *TTN* missense variants and *TTN*tv

Of the 47 VUS identified in the CTRCD cohort, 19 were *TTN* missense variants. Since the role of *TTN* missense variants in cardiac disease is still under debate, we assessed the yield of genetic testing without inclusion of *TTN* missense variants. After exclusion of the *TTN* missense variants, 19 (41.3%) CTRCD patients were identified with a VUS or likely pathogenic variant, compared to 27 (58.7%) DCM patients (*p* = 0.095) and 25 (22.5%) patients of the negative control cohort (*p* = 0.017; Fig. 1B). However*,* variants in *TTN*, including *TTN* missense variants were more prevalent in both DCM (71.7%; *p* < 0.001) and CTRCD patients (47.8%, *p* = 0.023) compared to the negative control cohort (28.8%). On an allelic level, omitting *TTN* missense variants resulted in a variant burden of 0.0039 in the negative control cohort, compared to 0.0058 in CTRCD patients (*p* = 0.092) and 0.0080 in DCM patients (*p* < 0.001).

As *TTN*tv were the only (likely) pathogenic variants within the CTRCD cohort, we assessed the difference in variant burden between CTRCD and DCM patient, without taking *TTN*tv and *TTN* missense variants into account. Only 34.8% of CTRCDC patients carried a non-TTN variant compared to 58.7% of DCM patients (*p* = 0.022), illustrating the importance of *TTN* variants in this population.

### Variant characteristics: prevalence and predictive scores

**Fig. 2:**
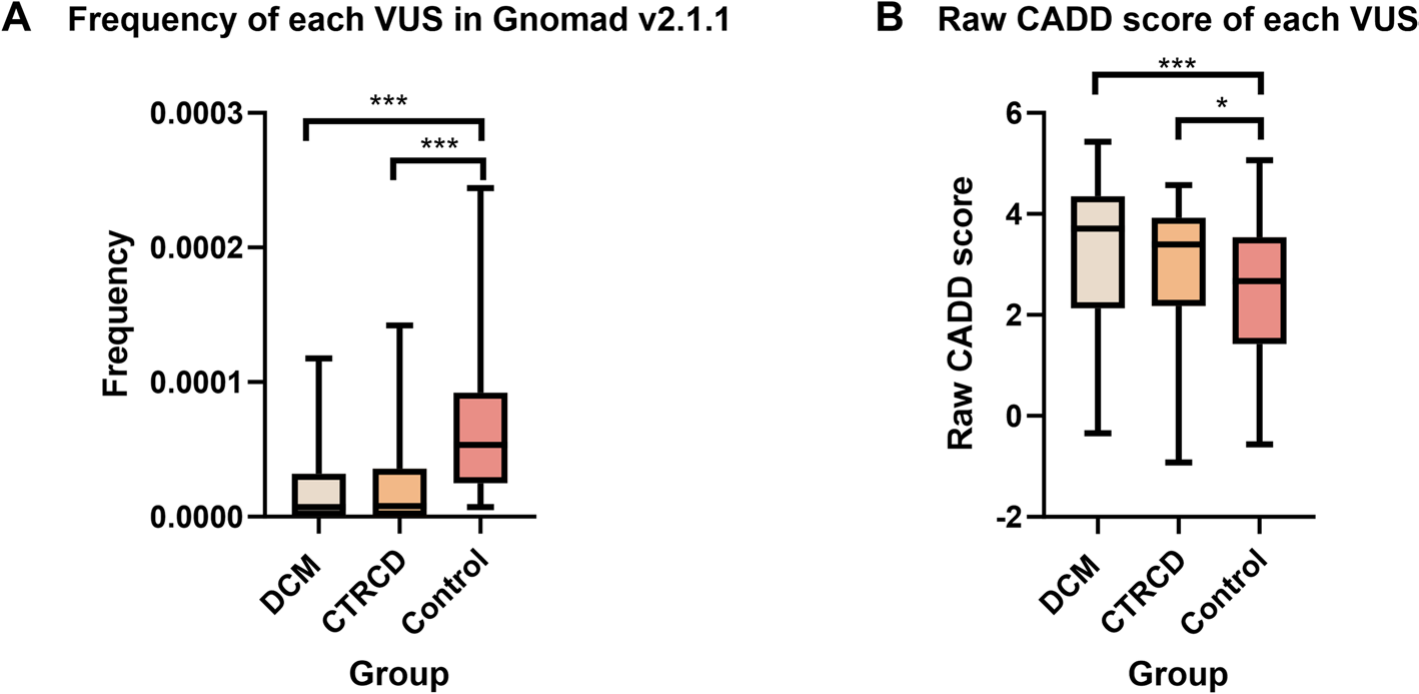
Frequency of the variants in GnomAD and raw CADD scores according to patient cohort. **A** For each specific VUS identified in our three cohorts, the frequency in the GnomAD population database was checked. The variants in the negative control cohort were significantly more frequent in GnomAD than those in the DCM and CTRCD patient cohorts. **B** For each specific variant identified in our three cohorts, the raw CADD-score was checked. The variants in the negative control cohort had significantly lower CADD scores than those in the DCM and CTRCD patient cohorts. The median of variants of each cohort are shown, the box displays the interquartile distance and whiskers display the minimal and maximal frequency. CADD: Combined Annotation Dependent Depletion; CTRCD: cancer therapy-related cardiac dysfunction; DCM: dilated cardiomyopathy; VUS: variant of uncertain significance

We compared the prevalence of all identified variants in a general population (GnomAD v2.1.1). The VUS in the negative control cohort were significantly more frequent in the GnomAD database compared to the VUS in the CTRCD and DCM cohort (Fig. 2). For all VUS the Combined Annotation Dependent Depletion (CADD) score was obtained. The CADD scores predict the deleteriousness of variants based on a combination of prediction scores and information on conservation, and a higher score means the variant is less likely to be benign [20, 21]. The VUS in the negative control cohort had significantly lower CADD scores than the VUS in the CTRCD and DCM cohort (Fig. 2).

### Definitive DCM genes

The initial analysis included a broad panel of 59 genes linked to both arrhythmogenic cardiomyopathy, DCM and hypertrophic cardiomyopathy. However, CTRCD most typically presents as cardiomyopathy with reduced LVEF, similar to DCM. We therefore repeated the analysis, only considering variants in genes classified as definitive DCM-gene (*BAG3, DES, FLNC, LMNA, MYH7, PLN, RBM20, SCN5A, TNNC1, TNNT2, TTN*) and genes with strong (*DSP*) or moderate evidence of involvement in DCM (*ACTC1, ACTN2, JPH2, NEXN, TNNI3, TPM1, VCL*) [22]. Genetic yield in these 19 genes in the different cohorts is displayed in Table 3. Overall, differences between the three groups were similar when the 19 selected genes or the whole 59 CM panel were considered.

**Table 3:** Overview of genetic yield of testing in 19 DCM genes

**Gene**	**CTRCD (*n* = 46)**	**DCM (*n* = 46)**	**Negative control cohort (*n* = 111)**	***P*-value**		
				**CTRCD vs DCM**	**CTRCD vs Control**	**DCM vs control**
**Definitive DCM gene**						
***BAG3***	0 (0%)	1 (2.2%)	1 (0.9%)	0.315	0.518	0.517
***DES***	0 (0%)	2 (4.3%)	0 (0%)	0.153	n.a	***0.027***
***FLNC***	2 (4.3%)	2 + 8 (21.7%)	3 (2.7%)	***0.013***	0.593	** < *0.001***
***LMNA***	0 (0%)	0 (0%)	1 (0.9%)	n.a	0.518	0.518
***MYH7***	1 (2.2%)	1 (2.2%)	0 (0%)	0.315	n.a	0.119
***PLN***	0 (0%)	0 (0%)	0 (0%)	n.a	n.a	n.a
***RBM20***	3 (6.5%)	0 (0%)	2 (1.8%)	0.078	0.125	0.360
***SCN5A***	0 (0%)	2 (4.3%)	2 (1.8%)	0.153	0.360	0.357
***TNNC1***	1 (2.2%)	0 (0%)	0 (0%)	0.315	0.119	n.a
***TNNT2***	1 (2.2%)	1 (2.2%)	0 (0%)	1.000	0.119	0.119
***TTN***	3 + 19 (47.8%)	3 + 30 (71.7%)	32 (28.8%)	***0.019***	***0.023***	** < *0.001***
**All definitive genes*:***	22 (47.8%)	25 (54.3%)	35 (31.5%)	0.532	0.053	***0.007***
**Genes with strong evidence of involvement in DCM**						
***DSP***	0 (0%)	1 + 1 (4.3%)	2 (1.8%)	0.153	0.360	0.357
**Genes with moderate evidence of involvement in DCM**						
***ACTC1***	0 (0%)	0 (0%)	0 (0%)	n.a	n.a	n.a
***ACTN2***	0 (0%)	0 (0%)	0 (0%)	n.a	n.a	n.a
***JPH2***	0 (0%)	1 (2.2%)	0 (0%)	0.315	n.a	0.119
***NEXN***	0 (0%)	1 (2.2%)	2 (1.8%)	0.315	0.360	0.877
***TNNI3***	1 (2.2%)	0 (0%)	0 (0%)	0.315	0.119	n.a
***TPM1***	0 (0%)	1 (2.2%)	0 (0%)	0.315	n.a	0.119
***VCL***	2 (4.3%)	1 (2.2%)	1 (0.9%)	0.557	0.151	0.517
**All moderate genes**	3 (6.5%)	3 (6.5%)	3 (2.7%)	1.000	0.256	0.256
**All 19 genes**	23 (50%)	26 (56.5%)	39 (35.1%)	0.315	0.119	***0.013***

Number of patients with a variant in each gene are displayed. In the combined yield each patient is only counted once (the total number of variants is higher than the number of combined variant harbouring individuals as some patients carry more than 1 variant)

*N.a*. not applicable due to no variants present

When looking at individual genes, an increased burden of *FLNC* variants was observed in DCM patients compared to the negative control cohort (21.7% vs 2.7%; *p* < 0.001) and the CTRCD cohort (4.3%; *p* = 0.013)*.*

### Impact of genetic diagnosis on family members and further approach

Three patients with CTRCD carried a likely pathogenic *TTNtv.* Twelve family members were screened, of whom eight (66.7%) were variant carriers. All the variant harbouring family members were asymptomatic at the time of genetic diagnosis. Genotype positive family members were on average 50 years old (± 18). Further cardiac evaluation identified mild left ventricular dysfunction in one patient (LVEF 47%). In comparison, in the DCM population sixteen family members from five different families of patients with (likely) pathogenic variants were screened. Eleven of them carried the familial variant of whom three family members with *TTN*tv (average age: 46 year ± 20 year). In the DCM families, three patients had a DCM phenotype (27.3% of genotype positive family members), of whom two were symptomatic at time of diagnosis.

In three of the sixteen CTRCD patients carrying a VUS combined clinical and genetic cascade screening was performed. Six family members were tested of whom two carried the variant. Both family members were asymptomatic and had normal cardiac function at the time of genetic diagnosis. In the DCM cohort, fourteen family members from six families were screened and five were variant carriers. Only one of these genotype positive family members had a cardiac phenotype.

## Discussion

In this single centre retrospective analysis we were able to identify a significantly higher burden of cardiomyopathy-related genetic variants in adult patients with AC-induced CTRCD, compared to a negative control population.

Recently, several reports have hinted at a role for structural cardiac variants in the development of CTRCD following AC, describing selected cases of CTRCD patients with identified genetic variants.

Firstly, Linschoten et. al. described two cases of severe symptomatic CTRCD (LVEF decline to < 20%) after AC therapy, who were both harbouring a truncating variant in *TTN* [6]. Thereafter, other case series and case reports described selected CTRCD patients with a severe evolution or the presence of a positive family history of DCM in whom pathogenic variants in *TTN, MYH7* and *TNNT2* were identified [5, 7–9].

Garcia-Pavia and colleagues described the genetic burden in a larger CTRCD population, the comparison of the diagnostic yield of genetic testing between CTRCD and dilated cardiomyopathy remains elusive. In the present work we showed that diagnostic yield of genetic testing does not differ significantly between DCM and CTRCD patients but differs significantly compared to a negative control cohort.

All (likely) pathogenic variants in the CTRCD cohort were truncating variants in *TTN (TTNtv),* which were absent from the negative control cohort. Prevalence was 6.5% in our CTRCD cohort which is higher than the prevalence of *TTNtv* in the general population, described before (0.5-3%) [10, 13]. The increased prevalence of *TTNtv* in our CTRCD patients is in line with the data of Garcia-Pavia and colleagues who described a prevalence of 7.5% [10]. *TTN*tv are a common cause of dilated cardiomyopathy (DCM), occurring in approximately 25% of familial DCM [13]. In our DCM cohort in this study the prevalence of *TTN*tv was 6.5%. Previous work from our group showed *TTNtv* in 26% of patients who received a heart transplant due to non-ischemic DCM [23]. *TTN*tv are also predisposing for peripartum cardiomyopathy and alcoholic cardiomyopathy, two acquired forms of DCM [24, 25]. These findings support the 'second-hit theory' in (acquired forms of) DCM, stating that an external trigger might be required to reveal *TTN*tv effects. CTRCD is now comprehensively approached as systolic dysfunction (based on LVEF or global longitudinal strain) or signs of damage (based on cardiac troponins or (NT-pro)BNP) [2]. In the past however, only severe systolic dysfunction, often combined with dilatation of the left ventricle, was recognized as cardiotoxicity due to anthracyclines, and this was generally seen as a form of acquired dilated cardiomyopathy, hence the comparison with a ‘possibly genetic’ DCM cohort in the current work [26].

Knowledge on which genes could be useful for screening in this population is still lacking. Analysis with a more selective panel of 19 DCM-associated genes showed comparable results to the analysis with the broader 59 gene panel. When looking at specific single genes, variants in *FLNC* were overrepresented in the DCM cohort compared to the negative control and CTRCD cohort. It is likely that *FLNC* variants have a higher overall penetrance and are less dependent on environmental factors to develop an overt phenotype. In contrast, variants in *TTN* showed a similar prevalence in CTRCD and DCM groups but were significantly enriched compared to the negative control group. This highlights the importance of 'second hits' in the setting of *TTN*tv. We cannot exclude that CTRCD patients with a *TTN*tv variant would have developed a DCM phenotype, even when not treated with anthracycline chemotherapy. Nonetheless, these results show that an underlying genetic predisposition is present in some CTRCD patients and that genetic screening in patient with CTRCD is warranted. CTRCD patients with an underlying variant had a tendency to earlier presentation than CTRCD patients without an underlying variant, but no difference in disease severity. The earlier presentation with an DCM phenotype, might be explained by a built-up of risk factors and strain on the heart throughout life, thereby passing a critical threshold and leading to a cardiac phenotype. Whereas patients without a variant might need more exposure to additional risk factors, even after exposure to AC, patients with a variant will reach this threshold sooner and could therefore present earlier.

Therapy-related risk factors increasing risk for CTRCD include concomitant treatment with radiotherapy (of the left chest) and/or trastuzumab. One could hypothesize that such ‘traditional’ risk factors are more frequent in patients without a genetic variant presenting with CTRCD. However, the current study was not powered to assess these differences.

In the setting of a ‘second-hit’ on a genetic vulnerable background, variants of unknown significance, may play a role as well. The assessment of prevalence of variants of uncertain significance (VUS) and *TTN* missense variants in CTRCD in our study are novel. 

Whether the current findings should lead to implementation of genetic screening *before* the start of AC in all patients, remains to be investigated. Ideally, a prospective study should be performed, assessing whether genetic screening, and subsequent adaptation of chemotherapeutic regimen, adapted follow-up and/or implementation of preventive measures, lead to improved outcomes in patients with a reduction of CTRCD and an increase in quality of life for patients. Possible measurements in high risk patients related to genetic susceptibility, can include the reduction of AC dose in genetic susceptible patients or a change in dosing regimen (with longer continuous administration) or the use of liposomal AC [1]. Additionally, follow-up can be adapted to be more regular and to include more modalities (including more frequent biomarker and/or echocardiographic assessment). Lastly, these patients at increased risk might be eligible for preventive treatment, with either classic heart-failure treatment, such as beta-blockade or inhibition of the renin-angiotensin-aldosterone axis, or with the only FDA- and EMA- approved, but expensive, preventive drug dexrazoxane [1].

Lastly, we performed (limited) cascade screening in family members of CTRCD patients harbouring a (likely) pathogenic variant. Despite the small sample size, we were able to identify eight family members at risk, of whom one had a decreased LVEF. The average age at time of genetic screening was 50 years and as a DCM phenotype can develop beyond this age, further follow-up of these family members is needed. Early identification of family members at risk allows for early diagnosis and initiation of treatment, before symptoms develop, which leads to better outcomes overall. This highlights the importance of genetic diagnosis in historical CTRCD patients.

The design and clinical use of a combined clinical, genetic and polygenetic risk score (taking both rare variants and more frequent single nucleotide polymorphisms into account) to stratify individuals before the start of AC treatment, seems feasible as this has been done in other cardiovascular diseases [27]. Of note, several studies have shown variation in genes with a role in the metabolism or transport of AC in patients with CTRCD [28, 29]. Both rare variants and single nucleotide polymorphisms seem to affect the pharmacogenomic CTRCD risk. For example, a large GWAS analysis revealed *PRDM2*, a transcription factor involved in repair of double strand DNA-breaks and oxidative stress, as a susceptible locus for LVEF decline after AC [30]. Some groups have even tried to include some of these pharmacogenomic risk factors in risk prediction scores [31–34]. This information is an additional piece to the unsolved puzzle of CTRCD but was outside the scope of the current work.

Some limitations of the current work should be addressed. Firstly, we did not include a cohort of patients with a prior history of cancer treated with anthracyclines who did *not* develop CTRCD. Although it is unlikely that a group of patients with cancer and the absence of cardiac disease would have a higher burden of variants in cardiomyopathy-related genes than the diverse control cohorts used in the current manuscript we cannot exclude this with certainty. Second, longitudinal follow-up was not performed systematically, and future development of cardiac dysfunction is not assessed. Overall, the current cohort is a rather small cohort, limiting the possibility for additional analyses on the influence of modifiers and additional risk factors.

## Conclusion

In patients with CTRCD an increased burden of genetic variants in cardiomyopathy genes is observed, similar to that of DCM patients. Variant harbouring status did not impact the severity of CTRCD, nor the rate of recovery after cessation of chemotherapy and start of heart failure treatment. But patients with a variant more frequently presented with early (< 1 year) CTRCD.

Since a definite genetic diagnosis can be made for a clinically important number of patients, with important consequences on familial screening, genetic testing should be considered in patients presenting with CTRCD.

### Supplementary Information

**Additional file 1: Supplementary Table 1.** Overview of genes included in panel. **Supplementary Table 2.** Overview and classification of all variants identified in the CTRCD and DCM patient cohorts.

## Data Availability

The datasets used and/or analysed during the current study are available from the corresponding author on reasonable request.

## Abbreviations

AC: Anthracyclines
ACMG: American College of Medical Genetics and Genomics
CM: Cardiomyopathy
CTRCD: Cancer therapy-related cardiac dysfunction
DCM: Dilated cardiomyopathy
ESC: European Society of Cardiology
ESMO: European Society for Medical Oncology
GEN ±: Genotype positive/negative
LVEF: Left Ventricular Ejection Fraction
TAAD: Thoracic Aorta Aneurysmatic Disease
*TTN*tv: Truncating variants in Titin gene
VUS: Variant of Unknown Significance
