# Supplementary material for: Variants in structural cardiac genes in patients with cancer therapy-related cardiac dysfunction after anthracycline chemotherapy: a case control study
Source: Cardiooncology. 2024 Apr 30;10:26. doi: 10.1186/s40959-024-00231-3 (PMC11059765; doi:10.1186/s40959-024-00231-3)
Supplement: Supplementary file 1 — Additional file 1: Supplementary Table 1. Overview of genes included in panel. Supplementary Table 2. Overview and classification of all variants identified in the CTRCD and DCM patient cohorts. [file 40959_2024_231_MOESM1_ESM.docx]

**Supplementary data:**

**Supplementary Table 1 Overview of genes included in panel.**

| **CM 59 gene panel** |
| --- |
| *ABCC9* (ENST00000261200; ENST00000261201 exon 38), *ACTC1* (ENST00000290378), *ACTN2* (ENST00000542672; ENST00000366578 exon 8), *ALPK3* (ENST00000258888), *ANKRD1* (ENST00000371697), *BAG3* (ENST00000369085), *CALR3* (ENST00000269881), *CAV3* (ENST00000343849), *CRYAB* (ENST00000526180; ENST00000524660 exon1; ENST00000533971 for exon 2), *CSRP3* (ENST00000265968), *CTNNA2* (ENST00000433211), *DES* (ENST00000373960), *DSC2* (ENST00000280904; ENST00000251081 exon 16), *DSG2* (ENST00000261590), *DSP* (ENST00000379802), *EMD* (*STA*) (ENST00000369842), *FHL1* (ENST00000394155; ENST00000370683 exon 1 and 6), *FLH2* (ENST00000409177); *FLNC* (ENST00000325888); *GLA* (ENST00000218516), *JUP* (ENST00000393931), *JPH2* (ENST00000372980), *LAMA4* (ENST00000230538), *LAMP2* (ENST00000371335; ENST00000434600 exon 9; ENST00000200639 exon 9), *LDB3* (ENST00000429277; ENST00000372066 exon 4; ENST00000372056 exon 8; ENST00000361373 exon 7), *LMNA* (ENST00000368300; ENST00000368297 exon 2 and 11), *MIB1* (ENST00000261537), *MYBPC3* (ENST00000545968), *MYH6* (ENST00000405093), *MYH7* (ENST00000355349), *MYL2* (ENST00000228841), *MYL3* (ENST00000292327), *MYOZ2* (ENST00000307128), *MYPN* (ENST00000358913), *NEBL* (ENST00000377122; ENST00000417816 exon 1,2,3 and 4), *NEXN* (ENST00000334785), *PKP2* (ENST00000070846), *PLN* (ENST00000357525), *PPA* (ENST00000341595), *PRDM16* (ENST00000270722), *PRKAG2* (ENST00000287878), *RAF1* (ENST00000442415); *RBM20* (ENST00000369519), *RY*R2 (ENST00000366574), *SCN5A* (ENST00000333535; ENST00000413689 exon 6), *SDHA* (ENST00000264932); *SGCD* (ENST00000337851, ENST00000517913), *SYNE1* (ENST00000367255); *TAZ* (ENST00000601016), *TCAP* (ENST00000309889), *TGFB3* (ENST00000238682), *TMEM43* (ENST00000306077), *TNNC1* (ENST00000232975), *TNNI3* (ENST00000344887), *TNNT2* (ENST00000236918), *TPM1* (ENST00000403994; ENST00000334895 exon 1 and 8; ENST00000559397 exon 2; ENST00000358278 exon 6; ENST00000288398 exon 9; ENST00000559556 exon 9), *TTN* (ENST00000589042; ENST00000360870 exon 46), *TTR* (ENST00000237014), *VCL* (ENST00000211998). |

**Supplementary table 2: Overview and classification of all variants identified in the CTRCD and DCM patient cohorts.**

| **Cohort** | ***Gene*** | **c.** | **p.** | **Classification** |
| --- | --- | --- | --- | --- |
| **CTRCD** | *TTN* | c.3867delT | p.Glu1290Asnfs*107 | **UVKL4** |
|  | *TTN* | c.6377G>A | p.Trp2126Ter | **UVKL4** |
|  | *TTN* | c.100342C>T | p.Arg33448* | **UVKL4** |
|  | *ALPK3* | c.1166G>T | p.Arg389Leu | UVKL3 |
|  | *ALPK3* | c.3644G>A | p.Arg1215His | UVKL3 |
|  | *FLNC* | c.1348G>A | p.Val450Met | UVKL3 |
|  | *FLNC* | c.2675T>C | p.Val892Ala | UVKL3 |
|  | *LAMA4* | c.1319C>T | p.Arg440Leu | UVKL3 |
|  | *LDB3* | c.998G>A | p.Arg333His | UVKL3 |
|  | *LDB3* | c.321+1G>A |  | UVKL3 |
|  | *MYH 7* | c.5779A>T | p.Ile1927Phe | UVKL3 |
|  | *MYH6* | c.3948C>A | p.Asp1316Glu | UVKL3 |
|  | *MYH6* | c.1750G>A | p.Ala584Thr | UVKL3 |
|  | *MYOZ2* | c.479C>T | p.Pro160Leu | UVKL3 |
|  | *PRDM16* | c.1961C>T | p.Ala654Val | UVKL3 |
|  | *PRDM16* | c.1984G>A | p.Val662Met | UVKL3 |
|  | *RAF1* | c.793A>G | p.Met265Val | UVKL3 |
|  | *RAF1* | c.859dupA | p.Arg287Lysfs*30 | UVKL3 |
|  | *RBM20* | c.2893G>A | p.Gly965Arg | UVKL3 |
|  | *RBM20* | c.1376C>T | p.Thr459Ile | UVKL3 |
|  | *RBM20* | c.3458A>C | p.Glu1153Ala | UVKL3 |
|  | *RYR2* | c.3140A>G | p.Lys1047Arg | UVKL3 |
|  | *RYR2* | c.12047T>A | p.Phe4016Tyr | UVKL3 |
|  | *TAZ* | c.704T>C | p.Ile235Thr | UVKL3 |
|  | *TAZ* | c.758G>A | p.Arg253Gln | UVKL3 |
|  | *TMEM43* | c.112G>A | p.Val38Met | UVKL3 |
|  | *TNNC1* | c.89delG | p.Gly30Alafs*15 | UVKL3 |
|  | *TNNI3* | c.337G>A | p.Asp113Asn | UVKL3 |
|  | *TNNT2* | c.185C>A | p.Ala62Glu | UVKL3 |
|  | *TTN* | c.88847T>A | p.Ile29616Asn | UVKL3*-TTN missense* |
|  | *TTN* | c.13817C>G | p.Pro4606Arg | UVKL3*-TTN missense* |
|  | *TTN* | c.29638G>A | p.Glu9880Lys | UVKL3*-TTN missense* |
|  | *TTN* | c.93323T>C | p.Ile31108Thr | UVKL3*-TTN missense* |
|  | *TTN* | c.87448A>T | p.Ile29150Leu | UVKL3 *-TTN missense* |
|  | *TTN* | c.3514C>A | p.Leu1172Ile | UVKL3*-TTN missense* |
|  | *TTN* | c.43690T>A | p.Ser14564Thr | UVKL3*-TTN missense* |
|  | *TTN* | c.63775G>A | p.Val21259Ile | UVKL3*-TTN missense* |
|  | *TTN* | c.100400T>G | p.Val33467Gly | UVKL3*-TTN missense* |
|  | *TTN* | c.11788G>A | p.Glu3930Lys | UVKL3*-TTN missense* |
|  | *TTN* | c.80263T>G | p.Phe26755Val | UVKL3*-TTN missense* |
|  | *TTN* | c.58196G>A | p.Arg19399Gln | UVKL3*-TTN missense* |
|  | *TTN* | c.4247G>A | p.Arg1416His | UVKL3*-TTN missense* |
|  | *TTN* | c.91477G>A | p.Glu30493Lys | UVKL3*-TTN missense* |
|  | *TTN* | c.39752C>A | p.Ala13251Asp | UVKL3*-TTN missense* |
|  | *TTN* | c.90227C>T | p.Thr30076Met | UVKL3*-TTN missense* |
|  | *TTN* | c.45369C>G | p.Ile15123Met | UVKL3 *-TTN missense* |
|  | *TTN* | c.6232C>T | p.Pro2078Ser | UVKL3 *-TTN missense* |
|  | *TTN* | c.15896G>A | p.Gly5329Asp | UVKL3 *-TTN missense* |
|  | *VCL* | c.1961A>C | p.Asn654Thr | UVKL3 |
|  | *VCL* | c.1961A>C | Asn654Thr | UVKL3 |
| **DCM** | *DSP* | c.7354C>T | p.Gln2452* | **UVKL5** |
|  | *FLNC* | c.6864_6867dupCGCT | p.Val2290Argfs*23 | **UVKL5** |
|  | *FLNC* | c.7931_7932delGG | p.Gly2644Alafs*36 | **UVKL5** |
|  | *PRDM16* | c.3460G>T | p.Glu1154* | **UVKL4** |
|  | *TTN* | c.82525C>T | p.Arg27509* | **UVKL4** |
|  | *TTN* | c.57331C>T | p.Arg19111* | **UVKL4** |
|  | *TTN* | c.83575_83579delAAAGT | p.Lys27859Valfs*2 | **UVKL4** |
|  | *ALPK3* | c.2971A>T | p.Thr991Ser | UVKL3 |
|  | *ALPK3* | c.1166G>T | p.Arg389Leu | UVKL3 |
|  | *ALPK3* | c.3722G>A | p.Arg1241His | UVKL3 |
|  | *ANKRD1* | c.222dupA | p.Leu75Thrfs*8 | UVKL3 |
|  | *BAG3* | c.1411G>A | p.Glu471Lys | UVKL3 |
|  | *CALR3* | c.217C>G | p.Arg73Gly | UVKL3 |
|  | *DES* | c.352C>T | p.Arg118Cys | UVKL3 |
|  | *DES* | c.832C>T | p.Arg278Trp | UVKL3 |
|  | *DSC2* | c.2381C>T | p.Ser794Leu | UVKL3 |
|  | *DSP* | c.4679A>G | p.Gln1560Arg | UVKL3 |
|  | *FLNC* | c.1682T>C | p.Phe561Ser | UVKL3 |
|  | *FLNC* | c.349A>G | p.Ile117Val | UVKL3 |
|  | *FLNC* | c.6074C>T | p.Thr2025Ile | UVKL3 |
|  | *FLNC* | c.3259A>C | p.Thr1087Pro | UVKL3 |
|  | *FLNC* | c.6049G>A | p.Val2017Met | UVKL3 |
|  | *FLNC* | c.3790G>A | p.Gly1264Ser | UVKL3 |
|  | *FLNC* | c.652G>A | p.Ala218Thr | UVKL3 |
|  | *FLNC* | c.1094A>G | p.Glu365Gly | UVKL3 |
|  | *JPH2* | c.1271C>T | p.Pro424Leu | UVKL3 |
|  | *JUP* | c.56C>T | p.Thr19Ile | UVKL3 |
|  | *LAMA4* | c.2568G>C | p.Lys856Asn | UVKL3 |
|  | *LAMA4* | c.4583G>A | p.Arg1528His | UVKL3 |
|  | *MYBPC3* | c.2030C>T | p.Pro677Leu | UVKL3 |
|  | *MYH7* | c.2052G>A | p.Met684Ile | UVKL3 |
|  | *MYL2* | c.431delC | p.Pro144Leufs*3 | UVKL3 |
|  | *NEBL* | c.1745 G>C | p.Arg582Thr | UVKL3 |
|  | *NEXN* | c.421C>T | p.Arg141Cys | UVKL3 |
|  | *PRDM16* | 2362A>T | p.Met788Leu | UVKL3 |
|  | *PRDM16* | c.862C>T | p.Arg288Trp | UVKL3 |
|  | *RYR2* | c.429A>G | p.Ile1432Val | UVKL3 |
|  | *RYR2* | c.7784A>G | p.Asp2595Gly | UVKL3 |
|  | *SCN5A* | c.4748G>A | p.Arg1583His | UVKL3 |
|  | *SCN5A* | c.6046G>A | p.Val2016Met | UVKL3 |
|  | *SDHA* | c.970G>A | p.Glu324Lys | UVKL3 |
|  | *TAZ* | c.779T>G | p.Val260Gly | UVKL3 |
|  | *TNNT2* | c.451delC | p.Arg151Glyfs*41 | UVKL3 |
|  | *TPM1* | c.251A>G | p.Asp84Gly | UVKL3 |
|  | *TTN* | c.44281+1G>A |  | UVKL3 |
|  | *TTN* | c.85115G>A | p.Gly28372Glu | UVKL3 -TTN missense |
|  | *TTN* | c.67429A>G | p.Ser22477Gly | UVKL3 -TTN missense |
|  | *TTN* | c.37450G>A | p.Glu12484Lys | UVKL3 -TTN missense |
|  | *TTN* | c.17027T>A | p.Ile5676Asn | UVKL3 -TTN missense |
|  | *TTN* | c.66590G>A | p.Arg22197Gln | UVKL3 -TTN missense |
|  | *TTN* | c.8434G>C | p.Val2812Leu | UVKL3 -TTN missense |
|  | *TTN* | c.3133G>A | p.Vel1045Met | UVKL3 -TTN missense |
|  | *TTN* | c.92782G>C | p.Asp30928His | UVKL3 -TTN missense |
|  | *TTN* | c.5264A>G | p.Asn1755Ser | UVKL3 -TTN missense |
|  | *TTN* | c.3010G>A | p.Glu1004Lys | UVKL3 -TTN missense |
|  | *TTN* | c.43690T>A | p.Ser14564Thr | UVKL3 -TTN missense |
|  | *TTN* | c.96230G>A | p.Arg32077Gln | UVKL3 -TTN missense |
|  | *TTN* | c.31837C>G | p.Pro10613Ala | UVKL3 -TTN missense |
|  | *TTN* | c.81671A>G | p.Asn27224Ser | UVKL3 -TTN missense |
|  | *TTN* | c.100400T>G | p.Val33467Gly | UVKL3 -TTN missense |
|  | *TTN* | c.11788G>A | p.Glu3930Lys | UVKL3 -TTN missense |
|  | *TTN* | c.39287C>G | p.Pro13096Arg | UVKL3 -TTN missense |
|  | *TTN* | c.80942G>A | p.Arg26981Gln | UVKL3 -TTN missense |
|  | *TTN* | c.14232C>A | p.Asp4744glu | UVKL3 -TTN missense |
|  | *TTN* | c.97030G>A | p.Gly32344Ser | UVKL3 -TTN missense |
|  | *TTN* | c.20156T>C | p.Ile6719Thr | UVKL3 -TTN missense |
|  | *TTN* | c.45724A>G | p.Arg15242Gly | UVKL3 -TTN missense |
|  | *TTN* | c.76279A>G | p.Ser25427Gly | UVKL3 -TTN missense |
|  | *TTN* | c.96718G>A | p.Glu32240Lys | UVKL3 -TTN missense |
|  | *TTN* | c.50714G>A | p.Arg16905His | UVKL3 -TTN missense |
|  | *TTN* | c.105920T>C | p.Val35307Ala | UVKL3 -TTN missense |
|  | *TTN* | c.83279A>T | p.ASn27760Ile | UVKL3 -TTN missense |
|  | *TTN* | c.13870C>T | p.His4624Tyr | UVKL3 -TTN missense |
|  | *TTN* | c.13437A>T | p.Arg4479Ser | UVKL3 -TTN missense |
|  | *TTN* | c.90104G>A | p.Arg30035His | UVKL3 -TTN missense |
|  | *VCL* | c.2197G>T | p.Asp733Tyr | UVKL3 |
| **Negative control cohort: TAAD** | *ABCC9* | c.1849G>A | p.Asp617Asn | UVKL3 |
|  | *ALPK3* | c.1688T>C | p.Met563Thr | UVKL3 |
|  | *ALPK3* | c.3302G>A | p.Gly1101Asp | UVKL3 |
|  | *BAG3* | c.230C>T | p.Pro77Leu | UVKL3 |
|  | *DSC2* | c.1721G>A | p.Ser574Asn | UVKL3 |
|  | *FLNC* | c.3022C>T | p.Arg1008Cys | UVKL3 |
|  | *JUP* | c.2069A>G | p.Asn690Ser | UVKL3 |
|  | *LMNA* | c.1567G>A | p.Gly523Arg | UVKL3 |
|  | *MYH6* | c.2398C>T | p.Arg800Cys | UVKL3 |
|  | *MYH6* | c.161G>A | p.Arg54Gln | UVKL3 |
|  | *MYPN* | c.2831C>T | p.Ala944Val | UVKL3 |
|  | *NEXN* | c.893C>G | p.Thr298Arg | UVKL3 |
|  | *PRDM16* | c.2780A>C | p.His927Pro | UVKL3 |
|  | *PRDM16* | c.2783A>C | p.His928PRo | UVKL3 |
|  | *PRDM16* | c.3221C>T | p.Ser1074Leu | UVKL3 |
|  | *RBM20* | c.1066A>C | p.Thr356Pro | UVKL3 |
|  | *RBM20* | c.2410G>A | p.Glu804Lys | UVKL3 |
|  | *RYR2* | c.1454G>A | p.Arg485Gln | UVKL3 |
|  | *SCN5A* | c.5479G>A | p.Ala1827Thr | UVKL3 |
|  | *TCAP* | c.337C>T | p.Leu113Phe | UVKL3 |
|  | *TCAP* | c.388C>T | p.Arg130Cys | UVKL3 |
|  | *TGFB3* | c.813G>C | p.Lys271Asn | UVKL3 |
|  | *TTN* | c.16328T>C | p.Val5443Ala | UVKL3 -TTN missense |
|  | *TTN* | c.53444T>C | p.Ile17815Thr | UVKL3 -TTN missense |
|  | *TTN* | c.84977G>A | p.Arg28326Gln | UVKL3 -TTN missense |
|  | *TTN* | c.5264A>G | p.Asn1755Ser | UVKL3 -TTN missense |
|  | *TTN* | c.37099C>G | p.Pro12367Ala | UVKL3 -TTN missense |
|  | *TTN* | c.21364G>A | p.Ala7122Thr | UVKL3 -TTN missense |
|  | *TTN* | c.57442A>G | p.Met19148Val | UVKL3 -TTN missense |
|  | *TTN* | c.3241G>A | p.Ala1081Thr | UVKL3 -TTN missense |
|  | *TTN* | c.83630G>A | p.Arg27877His | UVKL3 -TTN missense |
|  | *TTN* | c.85327G>A | p.Val28443Ile | UVKL3 -TTN missense |
|  | *TTN* | c.79226G>A | p.Arg26409His | UVKL3 -TTN missense |
|  | *TTN* | c.98021G>A | p.Arg32674His | UVKL3 -TTN missense |
|  | *TTN* | c.94553T>C | p.Val31518Ala | UVKL3 -TTN missense |
|  | *TTN* | c.91276C>T | p.Pro30426Ser | UVKL3 -TTN missense |
|  | *TTN* | c.26329G>A | p.Val8777Ile | UVKL3 -TTN missense |
|  | *TTN* | c.18550G>A | p.Ala6184Thr | UVKL3 -TTN missense |
|  | *TTN* | c.415C>T | p.Arg139Trp | UVKL3 -TTN missense |
|  | *TTN* | c.45247C>T | p.Arg15083Trp | UVKL3 -TTN missense |
|  | *VCL* | c.1237G>A | p.Ala413Thr | UVKL3 |
| **Negative control cohort: COVID** | *DSP* | c.137G>A | p.Gly46Asp | UVKL3 |
|  | *DSP* | c.5449G>A | p.Val1817Met | UVKL3 |
|  | *FLNC* | c.5252G>A | p.Arg1751His | UVKL3 |
|  | *FLNC* | c.5252G>A | p.Arg1751His | UVKL3 |
|  | *LAMA4* | c.1541G>A | p.Arg514Gln | UVKL3 |
|  | *LDB3* | c.1253C>G | p.Pro308Arg | UVKL3 |
|  | *MYBPC3* | c.787G>A | p.Gly263Arg | UVKL3 |
|  | *MYBPC3* | c.2063C>A | p.Thr688Lys | UVKL3 |
|  | *MYPN* | c.3544G>A | p.Gly1182Ser | UVKL3 |
|  | *NEXN* | c.1088C>G | p.Thr363Arg | UVKL3 |
|  | *PRDM16* | c.2503G>A | p.Ala835Thr | UVKL3 |
|  | *RYR2* | c.8145G>T | p.Glu2715Asp | UVKL3 |
|  | *SCN5A* | c.393-5C>A |  | UVKL3 |
|  | *TTN* | c.42524T>G | p.Phe14175Cys | UVKL3 -*TTN* missense |
|  | *TTN* | c.36936A>T | p.Lys12312Asn | UVKL3 -*TTN* missense |
|  | *TTN* | c.15227C>T | p.Pro5076Leu alternative exon 46 | UVKL3 -*TTN* missense |
|  | *TTN* | c.56872G>C | p.Asp18958His | UVKL3 -*TTN* missense |
|  | *TTN* | c.36982A>G | p.Thr12328Ala | UVKL3 -*TTN* missense |
|  | *TTN* | c.15302A>G | p. Glu5101Gly alternative exon 46 | UVKL3 -*TTN* missense |
|  | *TTN* | c.82489C>A | p.Pro27497Thr | UVKL3 -*TTN* missense |
|  | *TTN* | c.38902C>T | p.Pro12968Ser | UVKL3 -*TTN* missense |
|  | *TTN* | c.13976A>G | p.Tyr4659Cys | UVKL3 -*TTN* missense |
|  | *TTN* | c.93581A>G | p. Tyr31194Cys | UVKL3 -*TTN* missense |
|  | *TTN* | c.36982A>G | p.Thr12328Ala | UVKL3 -*TTN* missense |
|  | *TTN* | c.45599C>G | p.Ala15200Gly | UVKL3 -*TTN* missense |
|  | *TTN* | c.98716G>A | p. Val32906Ile | UVKL3 -*TTN* missense |
|  | *TTN* | c.72766A>G | p.Asn24256Asp | UVKL3 -*TTN* missense |
